# A Periplasmic Antimicrobial Peptide-Binding Protein Is Required for Stress Survival in *Vibrio cholerae*

**DOI:** 10.3389/fmicb.2019.00161

**Published:** 2019-02-05

**Authors:** Jessica Saul-McBeth, Jyl S. Matson

**Affiliations:** Department of Medical Microbiology and Immunology, University of Toledo, Toledo, OH, United States

**Keywords:** *Vibrio cholerae*, cholera, stress response, antimicrobial peptides, outer membrane proteins

## Abstract

*Vibrio cholerae* must sense and respond appropriately to stresses encountered in the aquatic environment and the human host. One stress encountered in both environments is exposure to antimicrobial peptides (AMPs), produced as a part of the innate immune response by all multicellular organisms. Previous transcriptomic analysis demonstrated that expression of Stress-inducible protein A (SipA) (VCA0732), a hypothetical protein, was highly induced by AMP exposure and was dependent on a specific uncharacterized two-component system. In order to better understand role of this protein in stress relief, we examined whether it shared any of the phenotypes reported for its homologs. SipA is required for survival in the presence of two other stressors, cadmium chloride and hydrogen peroxide, and it localizes to the bacterial periplasm, similar to its homologs. We also found that SipA physically interacts with OmpA. Importantly, we found that SipA binds AMPs in the bacterial periplasm. This suggests a model where SipA may act as a molecular chaperone, binding AMPs that enter the periplasm and delivering them to OmpA for removal from the cell. While El Tor *V. cholerae* strains lacking SipA do not show a survival defect in the presence of AMPs, we found that Classical *sipA* mutants are less able to survive in the presence of AMPs. This phenotype is likely masked in the El Tor background due to a functional lipid A modification system that increases AMP resistance in these strains. In summary, we have identified a protein that contributes to a novel mechanism of stress relief in *V. cholerae*.

## Introduction

Pathogenic bacteria have evolved to survive in diverse hosts and environments. In order to thrive in these different niches, many bacteria have developed sophisticated mechanisms to alter specific gene expression, resulting in adaptation and survival in the presence of stress. One example of a successful pathogen is *Vibrio cholerae*, a Gram-negative bacterium that causes the diarrheal disease cholera. Not only is *V. cholerae* able to alter its gene expression quickly in response to the aquatic environment, with fluctuating salinity, pH, and nutrient availability, but it can also survive stresses in the human gut, where it may encounter reactive oxidative species, competing commensal bacteria, and other stressors (Joelsson et al., 2007). One of the stresses *V. cholerae* must overcome to successfully colonize the human intestine is antimicrobial peptides (AMPs) produced by intestinal epithelial cells as a part of innate immunity.

Antimicrobial peptides are small, positively charged peptides that have both hydrophobic and hydrophilic residues near the N-termini, which enable them to interact with both the aqueous environment and lipid-rich membranes (Izadpanah and Gallo, 2005). AMPs are produced by bacteria, archeaea, and eukarya (including mammals), and are able to either lyse or prevent replication of a broad spectrum of organisms including bacteria, viruses, and parasites (Bahar and Ren, 2013). There are two main groups of AMPs produced by the human host: cathelicidins and defensins. LL-37, the only human member of the cathelicidin family, contains an N-terminal signal peptide and can function both as an antimicrobial and as a chemoattractant (Durr et al., 2006). Polymyxin B, another example of a cationic AMP, is derived from the soil bacterium *Bacillus polymyxa* (Brownlee and Jones, 1948). Historically, polymyxin B has been used to clinically differentiate the Classical and El Tor biotypes of *V. cholerae*, and has also been widely used in studies on bacterial stress. Many AMPs, including LL-37 and polymyxin B, are thought to act on Gram-negative bacteria by making initial non-specific interactions with the membrane through electrostatic interactions with the negatively charged lipopolysaccharide (LPS) layer (Izadpanah and Gallo, 2005; Sorensen et al., 2008). Due to their amphipathic structure, cationic AMPs can enter the membrane by forming pores or channels (Izadpanah and Gallo, 2005). Once inside the cell, through not clearly defined mechanisms, both polymyxin B and LL-37 can enter the periplasm and cytoplasm where they may interfere with DNA and protein synthesis (Choi et al., 2017).

Due to the potential lethality of AMPs, *V. cholerae* has developed a range of strategies to overcome AMP damage including: the production of outer membrane vesicles (OMVs) to titrate the AMPs, changes in porin composition of the outer membrane to modify permeability, the use of efflux pumps to export AMPs, LPS modifications to prevent AMP binding, and two-component systems (TCS) that transcriptionally regulate all of these mechanisms (Nizet, 2006; McBroom and Kuehn, 2007; Band and Weiss, 2015). In *V. cholerae* and other Gram-negative bacteria, the outer surface is composed of LPS, which acts as the first line of defense against extracellular stresses, including AMPs. Components of the LPS, particularly the lipid A and core oligosaccharide, are negatively charged, allowing AMPs to bind easily. However, *V. cholerae* encodes a lipid A modification system that alters the charge of the LPS, reducing AMP binding and subsequent entry into the bacterial cell. This modification system is encoded by the *almEFG* gene cluster, and alters the LPS through addition of a glycine or diglycine to lipid A (Henderson et al., 2014; Herrera et al., 2014). Interestingly, Classical strains of *V. cholerae* contain a frameshift mutation in *almF*, resulting in a non-functional modification system (Henderson et al., 2014). This explains why classical *V. cholerae* strains are far more sensitive to AMPs than El Tor strains (Matson et al., 2010; Henderson et al., 2014, 2017).

Two-component systems are important regulators of stress response mechanisms in bacteria. They are composed of a sensor kinase, located in the inner membrane, that senses a change in the external environment, and the response regulator, located in the cytoplasm, that responds accordingly by activating a specific set of genes. *V. cholerae* encodes TCSs that respond to AMP stress, including CarRS (VprAB) (Herrera et al., 2010, 2014; Bilecen et al., 2015; Henderson et al., 2017). In a previous study, we aimed to determine the function of a putative TCS (VC1638/39) that resembles PhoPQ in *Salmonella enterica* sv. Typhimurium in domain structure and conserved residues (Matson et al., 2017). In that study, RNA-Seq was used to identify genes differentially regulated by this TCS in the presence and absence of sublethal concentrations of polymyxin B (Matson et al., 2017). One of the interesting results concerned VCA0732, a 15 kDa hypothetical protein that belongs to a subfamily of the bacterial OB-fold family of proteins (Ginalski et al., 2004). Bacterial OB-fold proteins are characterized by a conserved oligonucleotide/oligosaccharide-binding (OB) fold domain which forms a binding pocket and is predicted to bind positively charged ligands and small molecules (Ginalski et al., 2004). We found that polymyxin B exposure highly induces *vca0732* expression, and that VC1638 and VC1639 are required for its expression (Matson et al., 2017). We hypothesized that VCA0732 must play a role in responding to the stress of AMP exposure and that the TCS was required to regulate this response. Puzzlingly, a *vca0732* mutant strain showed no survival defect in the presence of polymyxin B, making the role of VCA0732 in the AMP stress response unclear.

In the present study, we aimed to better understand the function of VCA0732 and to characterize its role in responding to stress in *V. cholerae*. We first examined whether or not VCA0732 functioned similarly to its known homologs in other bacteria. We found that VCA0732 has several similarities to its homologs, including periplasmic localization and its role in relieving stress caused by other molecules. VCA0732 also interacts with an outer membrane protein, OmpA, similar to its homologs. In addition, we found that VCA0732 directly binds AMPs both *in vivo* and *in vitro*. This suggests a model where VCA0732 (and its homologs) may function to chaperone AMPs to porins, positioning them for efflux out of the cell. While a *vca0732* mutant is insensitive to AMP killing in El Tor *V. cholerae*, we found that Classical strains require VCA0732 for survival. This suggests that the presence of the lipid A modification system in El Tor strains masks the survival phenotype observed in the Classical background. Taken together, these findings demonstrate that VCA0732 is a part of a previously unappreciated stress response mechanism and that it plays an important role in protecting *V. cholerae* from AMP killing. Due to the observed roles of VCA0732 in responding to AMPs and other stressors, we propose that it be named Stress-inducible protein A (SipA).

## Materials and Methods

### Bacterial Strains and Growth Conditions

All bacterial strains were grown at 37°C with agitation in Lysogeny Broth (LB) or on LB agar plates. Strains and plasmids used in this study are listed in Supplementary Table S1. Strains were derived from *V. cholerae* C6706 and O395 or *E. coli* K-12 strain BW25113. Plasmids used in this study were pKAS32, pTL61T, pBAD18-Kan and pBAD18-Cam (Linn and St Pierre, 1990; Guzman et al., 1995; Skorupski and Taylor, 1996). Antibiotics were used at the following concentrations: polymyxin B, 40 μg/mL for El Tor and 10 μg/mL for Classical; LL-37, 10 μg/mL for El Tor and 5 μg/mL for Classical; streptomycin, 100 μg/mL; kanamycin, 50 μg/mL; ampicillin, 100 μg/mL; chloramphenicol, 5 μg/mL. Arabinose was used at 0.2% to induce expression from the pBAD plasmids.

### Strain Construction

The C6706 *ΔsipA* (*Δvca0732*) strain used in this study has been described previously (Matson et al., 2017). The chromosomally His-tagged SipA strain was constructed using SOE-ing PCR (Innis, 1990). Briefly, 500 bp upstream and downstream of the *sipA* coding region were amplified separately using primers containing a 6× His sequence prior to the stop codon. After SOE-ing, the resulting PCR product was digested with *XbaI* and *SacI* and ligated into the suicide plasmid pKAS32 (Skorupski and Taylor, 1996). The resulting recombinant plasmid was transformed into *E. coli* SM10λpir. This strain was then mated with *V. cholerae* strain C6706. Integration of the plasmid into the *V. cholerae* chromosome was selected for by plating on TCBS (thiosulfate-citrate-bile-sucrose, Difco) containing 50 μg/mL ampicillin (Matson and DiRita, 2005) and conjugation was performed as previously described (Beck et al., 2004). O395*ΔsipA* and *ΔompA* were constructed by amplifying 500 bp segments upstream and downstream of the coding regions containing a internal deletion of the *sipA* or *ompA* gene. The products were joined together via SOE-ing PCR, cloned into suicide vector pKAS32, and transformed into *E. coli* as above (Skorupski and Taylor, 1996). The constructs were then conjugated into O395 *V. cholerae* as above and isolates containing the gene deletion were identified using PCR.

### Plasmid Construction

FLAG-tagged Crp, OmpA, and Tsp constructs were generated by amplification of the coding region of each gene from *V. cholerae* C6706 (Crp, OmpA) or O395 (Tsp) chromosomal DNA, using primers that added the C-terminal FLAG sequence with Radiant HiFi Ultra polymerase (Alkali Scientific Inc.). The coding sequence of *ygiW* was amplified from *E. coli* K-12. The *sipA* coding sequence was amplified from C6706. After amplification, the *ygiW, ompA*, and *sipA* PCR products were digested with *SacI* and *XbaI* and ligated into the arabinose-inducible expression vector pBAD18-Kan (Guzman et al., 1995). The *crp* construct was digested with *SacI* and *XbaI* and ligated into pBAD18-Cam. All constructs were confirmed by sequencing.

### Protein Purification

Proteins were purified according to Huntley et al. (2007) with a few modifications. *E. coli* strain JM101 containing plasmid constructs encoding SipA-6×his or VC1638-6×his was grown in LB to an optical density (OD) of ∼0.4, after which protein expression was induced for 4 h by the addition of 0.2% arabinose. Following induction, cells were collected by centrifugation and frozen overnight at -20°C. The following day, pellets were thawed and resuspended in 30 mL of 10 mM Tris, 500 mM NaCl, 10 mM imidazole, and complete mini EDTA free protease inhibitors (Qiagen). The cell suspension was freeze-thawed 3×, followed by removal of cellular debris by centrifugation. The resulting supernatant was applied to a column of pre-equilibrated HisPur Ni-NTA Resin (Thermo Fisher Scientific) followed by washing. Purified recombinant proteins were then eluted using lysis buffer containing 200 mM imidazole and dialyzed into 10 mM Tris, 100 mM NaCl, and 20% glycerol. Protein quantification was performed using a Qubit 2.0 fluorometer (Invitrogen).

### Enzyme-Linked Immunosorbent Assay (ELISA)

Ninety-six-well plates were coated with purified SipA or VC1638, at a concentration of 10 μg/mL overnight at 4°C in binding buffer (30 mM Na_2_CO_3_, 69 mM NAHCO_3_) (Lin et al., 2015). Plates were washed with PBS-T 3× and blocked for 1 h in 5% bovine serum albumin (BSA) + PBS-T at RT. Plates were washed and either LL-37 or biotinylated polymyxin B were added at 10 μg/mL and incubated for 1 h. Plates were washed again and anti-LL-37 (mouse; Santa-Cruz Biotechnology), was added for 1 h. After washing, either Goat pAb to Mouse IgG + IgM HRP (Abcam), or Strepavidin-HRP (Abcam) was added. TMB (Tetramethylbenzidine; Fisher Scientific) was used for detection and the reaction was stopped using 2N HCl. The colorimetric change was measured at OD_450_. Experiments were performed in triplicate and *P* values represent one experiment containing technical triplicates. Significance was determined using ANOVA with Tukey’s *post hoc* test.

### Survival Assays

Bacterial cultures were grown to an OD_600_ of ∼0.5 prior to adding cadmium chloride, hydrogen peroxide, polymyxin B, or LL-37. Aliquots of 900 μL of cells were incubated with 100 μL freshly prepared hydrogen peroxide at 2 μM for *V. cholerae* and 34 mM for *E. coli* for 15 min (Lee et al., 2010). Cadmium chloride was used at 200 μM for *E. coli* and 150 μM for *V. cholerae* for 40 min. Classical *V. cholerae* was treated with 10 μg/mL polymyxin B or 5 μg/mL LL-37 for 1 h. After treatment, samples were serially diluted and plated for enumeration. The percent survival was determined by dividing the CFU of treated samples by those of untreated samples × 100. Assays were performed in triplicate and *P* values were calculated from all three experiments using ANOVA with Tukey’s *post hoc* test.

### β-Galactosidase Assays

*Vibrio cholerae* strains were grown overnight at 37°C, then subcultured 1:50 into LB and grown for 4 h with aeration. Samples were placed on ice and chloramphenicol was added at 0.5 mg/mL. β-galactosidase assays were performed according to Miller (1972).

### Cellular Fractionation and Immunodetection

Bacterial fractionation was performed according to Petiti et al. (2017) with a few modifications. C6706, C6706 containing Tsp-FLAG or CRP-FLAG, or C6706 containing chromosomally His-tagged SipA was grown for 3 h with aeration. Polymyxin B was added at 40 μg/mL to induce SipA expression for 1 h. 0.2% arabinose was added to induce expression of Tsp-FLAG and CRP-FLAG. Whole-cell lysates were prepared by centrifugation and suspension of the pellet in 500 μL of Radioimmunoprecipitation assay buffer (RIPA) (25 mM Tris pH 8.0, 150 mM NaCl, 0.1% SDS, 0.5% sodium deoxycholate, 1% NP-40, and 10 mM PMSF). The lysate was then subjected to 3 freeze-thaw cycles, followed by centrifugation and collection of 200 μL of supernatant. The periplasmic fraction was isolated by centrifuging bacterial cells, washing 3× with 1× PBS, and suspension of the pellet by inversion in 200 μL TES buffer (0.5 M sucrose, 0.5 mM EDTA, 200 mM Tris–HCl, pH 8.0). After careful mixing, 720 μL 2× diluted TES buffer containing 10 mg/mL lysozyme was added and incubated on ice for 30 min. Following centrifugation for 30 min at max speed, 200 μL of the supernatant was collected, representing the periplasmic fraction. The pellet was resuspended in 720 μL of 2× diluted TES buffer containing 2 mM MgCl_2_ and lysed by 3× freeze-thaw cycles, followed by centrifugation and collection of the supernatant. The supernatant was then centrifuged at 100,000 × *g* for 45 min and 200 μL of supernatant was collected, representing the cytoplasmic fraction. All fractions were precipitated with 10% TCA for 30 min on ice. Samples were washed with 1 mL acetone, centrifuged, resuspended in SDS–loading buffer, and boiled for 5 min. Proteins were separated using 15% (wt/vol) polyacrylamide gels, transferred to nitrocellulose membranes and probed with either His HRP-labeled Mouse Monoclonal IgG_1_ (R&D Systems) or OctA-Probe (H-5) HRP (Santa-Cruz Biotechnology). The protein fractionation controls used were: Tsp-FLAG (periplasm) and CRP-FLAG (cytoplasm).

### Outer Membrane Protein (OMP) Isolation (ROMP)

Outer membrane proteins were isolated via rapid outer membrane protein procedure (ROMP) (Carlone et al., 1986). Strains containing recombinant protein were grown and induced for 4 h. After growth, samples were centrifuged and pellets were resuspended in 1 mL 10 mM HEPES, pH 7.4. Lysis was performed by repeated freeze-thaw cycles. Cellular debris was removed by centrifugation at 15,600 × *g* for 5min at 4°C. The supernatant was then centrifuged at 15,600 × *g* at 4°C for 30 min to pellet the cell membrane fraction. The supernatant was removed and the membrane-containing pellet was resuspended in 0.2 mL 10 mM HEPES, pH 7.4. The membranes were solubilized by the addition of 0.2 mL 2% sarkosyl in 10 mM HEPES, pH 7.4, and incubated on ice for 30 min with intermittent pipetting. The outer membrane was then pelleted by centrifugation as described above and washed with 10 mM HEPES. After washing, the pellet was resuspended in SDS-PAGE loading buffer.

### Far-Western Blotting

Outer membrane protein fractions from C6706 expressing OmpA-FLAG were serially diluted and separated by SDS-PAGE. The far western protocol is based on Chen, et al. with modifications (Wu et al., 2007). After separation by electrophoresis, the proteins were transferred to a nitrocellulose membrane (GE Healthcare) and the membrane was blocked in 5% milk in PBST for 1 h. The membrane was then incubated with purified His-tagged SipA at 10 μg/mL overnight at 4°C. After incubation, SipA was detected via His HRP-labeled Mouse Monoclonal IgG_1_ (R&D Systems).

### Immunoprecipitation

C6706 or C6706*ΔsipA* containing a SipA plasmid was grown for 3 h in LB with shaking, and 10 μg/mL LL-37 was added for 1 h before pelleting by centrifugation. The pellet was then resuspended in 500 μL 1× PBS containing 1 mM DSP (Dithiobis Succinimidyl Propionate; Pierce) to crosslink proteins, and incubated on ice for 30 min. The reactions were quenched by adding 50 mM Tris–HCl, pH 8.0 for 15min on ice, and the samples were centrifuged at 4°C. The resulting pellet was resuspended in 500 μL RIPA buffer and subjected to 3× freeze-thaw lysis. After centrifugation at 4°C, 50 μL of supernatant was collected and used as the input control. The remaining 450 μL of supernatant was added to 40 μL HisPur Ni-NTA equilibrated in RIPA buffer and rotated at 4°C for 1 h. Samples were centrifuged at 2000 × *g* for 2 min and washed in RIPA buffer 3× before resuspending in SDS–loading buffer and boiling for 15 min. SDS-PAGE and western blotting for LL-37 and SipA-His were performed as above. For identification of potential SipA interaction partners, C6706 containing a SipA plasmid was grown, crosslinked, and immunoprecipitated in the same manner (in the absence of LL-37). Proteins in the elution were visualized by Coomassie staining, excised from the gel, and identified by mass spectrometry (MS Bioworks, Ann Arbor, MI, United States).

Strains containing both the OmpA-FLAG and SipA-6× his constructs, or OmpA-FLAG construct and empty vector, were grown and protein expression was induced for 1 h at 37°C. The cells were centrifuged and crosslinked with DSP as above. Cells were lysed in buffer containing 0.5M HEPES, 2M MgCl_2_, 10 mL 1 M KCl, 10% glycerol, 1% Triton-X, and 20 mM imidazole (Wu et al., 2007) by 3× freeze-thaw. Cellular debris was removed by centrifugation and the supernatant was added to HisPur Ni-NTA beads and rotated overnight at 4°C. Immunoprecipitation and western blotting were performed as above.

## Results

### A *V. cholerae sipA* Mutant Is Sensitive to Cadmium Chloride and Hydrogen Peroxide Stress

**FIGURE 1:**
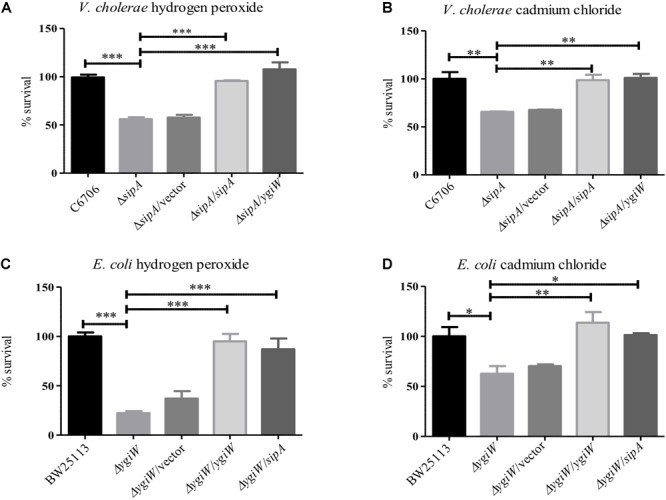
SipA is required for survival in the presence of cadmium chloride and hydrogen peroxide. *V. cholerae* **(A,B)** and *E. coli* **(C,D)** were grown to mid-logarithmic phase and then treated with: **(A)** 2 μM hydrogen peroxide for 15 min, **(B)** 150 μM cadmium chloride for 45 min, **(C)** 34 mM hydrogen peroxide for 15 min, and **(D)** 200 μM cadmium chloride for 45 min. After treatment, serial dilutions of each culture were plated for enumeration. Data represents the means and standard deviations of at least three samples. ^∗^*p* = < 0.05, ^∗∗^*p* = < 0.01, ^∗∗∗^*p* < 0.001.

To understand the role of SipA in stress resistance, we wanted to determine if it shared any of the known phenotypes or functions with its homolog from *Escherichia coli*, YgiW (Lee et al., 2010). Since YgiW is required for survival in the presence of cadmium chloride and hydrogen peroxide stress (Lee et al., 2010), we examined the survival of an El Tor *V. cholerae sipA* deletion strain in the presence of these compounds. We found that when exposed to hydrogen peroxide, a *sipA* mutant strain showed a significant defect in survival (Figure 1A). We saw a similar survival defect in the presence of cadmium chloride (Figure 1B). Expression of SipA from a plasmid was able to restore wild type resistance to both compounds. In addition, we also tested whether the *ygiW* gene from *E. coli* could complement these phenotypes in the *V. cholerae* background, and found that it also restored survival to wild type levels (Figure 1A,B). Finally, we obtained a *ygiW* mutant *E. coli* strain [Keio collection (Baba et al., 2006)] and tested both *ygiW* and *sipA* for complementation in the *E. coli* background. Again, both homologs were able to restore resistance to hydrogen peroxide and cadmium chloride to wild type levels (Figure 1C,D). Overall, these results show that both *ygiW* and *sipA* complement and cross-complement the resistance phenotypes in both bacterial species (Figure 1A–D). This suggests that SipA in *V. cholerae* plays a role in stress relief and has a similar cellular function as YgiW in *E. coli* under the conditions tested.

Our previous work showed that AMP stress induces expression of *sipA* (Matson et al., 2017). We therefore wanted to determine if these two other stressors also induce its expression. A *sipA-lacZ* reporter fusion was used to assess *sipA* promoter activity in the presence of cadmium chloride and hydrogen peroxide. While both compounds clearly induced *sipA* expression in *V. cholerae*, it was to a lower level than the induction by polymyxin B (Supplementary Figure S1).

### SipA Localizes to the Periplasm

**FIGURE 2:**
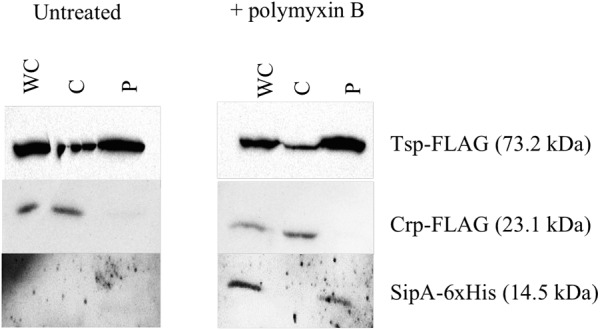
SipA localizes to the periplasmic space. Immunoblotting of whole-cell lysates (WC) and soluble fractions from a *V. cholerae* strain containing a chromosomal 6×his-tagged *sipA*. The membrane was probed with anti-FLAG and anti-His antibodies. Tsp-FLAG and Crp-FLAG were used as markers for the periplasmic (P) and cytoplasmic (C) fractions, respectively.

The known homologs of SipA, YgiW (*E. coli*) and YdeI (*Salmonella enterica*), localize to the periplasmic space (Ginalski et al., 2004; Pilonieta et al., 2009). In addition, subcellular localization prediction tools suggest that SipA is a periplasmic protein (data not shown). In order to determine whether SipA localizes to the periplasm of *V. cholerae* we performed cellular fractionation studies. These studies were performed in the presence and absence of polymyxin B in order to determine whether or not the antimicrobial peptide influences localization of the protein, in addition to its established role in *sipA* expression (Matson et al., 2017). We first generated a strain of *V. cholerae* where *sipA* was chromosomally His-tagged. This allowed us to detect SipA using anti-His antiserum, and avoid any localization artifacts that could be introduced by overexpression of the protein. We co-expressed FLAG-tagged Tsp and FLAG-tagged CRP in this background to use as periplasmic and cytoplasmic control proteins, respectively. Tail-specific protease (Tsp) is a periplasmic protease that degrades the virulence regulator TcpP when conditions do not favor virulence gene expression (Teoh et al., 2015). CRP (cAMP receptor protein) is a cytoplasmic protein that functions as a global transcriptional regulator. Upon separation into the cytoplasmic and periplasmic fractions, the majority of SipA localized to the periplasmic compartment, similar to the known periplasmic protein, Tsp (Figure 2). In the absence of polymyxin B, too little SipA was expressed for detection of the protein, consistent with the previous observation that its expression is highly induced by polymyxin B exposure (Matson et al., 2017). This demonstration that SipA localizes to the periplasmic space, similar to its homologs, suggests that its functional role occurs in that cellular compartment.

### SipA Physically Interacts With OmpA

**FIGURE 3:**
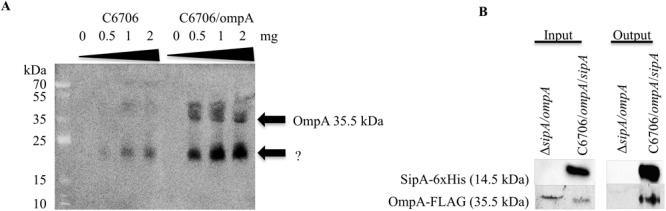
SipA interacts with OmpA. **(A)** C6706 with and without overexpressed OmpA was grown to an OD_600_ of 0.5. Outer membranes were isolated, and a range of isolated total outer membrane proteins (0, 0.5, 1, and 2 mg) was separated by SDS-PAGE and transferred to a membrane. Purified 6×his-SipA was incubated with the membrane overnight and detected using anti-6×His. Results are representative of at least three independent experiments. **(B)** *sipA* mutant *V. cholerae* overexpressing OmpA-FLAG and C6706 overexpressing OmpA-FLAG and SipA-6×His were grown to an OD_600_ of 0.5. Samples were crosslinked with 1 mM DSP and coimmunoprecipitation was performed via Ni-NTA beads. Samples were analyzed by SDS-PAGE and western blotting and detected using anti-FLAG and anti-His antibodies.

Previous studies on the SipA homologs showed that they interact with outer membrane proteins (OMPs). YgiW interacts with OmpF in *E. coli*, while YdeI interacts with OmpD in *S. enterica* (Pilonieta et al., 2009). Therefore, we were interested in determining if SipA also interacted with an OMP in *V. cholerae*. Initially, to identify potential interaction partners, SipA was overexpressed from a plasmid and used to coimmunoprecipitate proteins after crosslinking. Bound proteins were identified using mass spectrometry. Although other studies hypothesized that SipA may interact with OmpU (Pilonieta et al., 2009), no OmpU fragments were identified in this pilot study. However, OmpA fragments were found (data not shown). To determine if SipA physically interacts with OmpA, we first performed far-western analysis. *V. cholerae* overexpressing FLAG-tagged OmpA was fractionated to isolate the outer membrane (OM) fraction. Increasing concentrations of OM fraction were separated by SDS-PAGE, transferred to a nitrocellulose membrane, and probed with purified His-tagged SipA. After incubation, bound SipA was detected using anti-His antibodies. We also performed an identical experiment in wild type cells expressing native levels of OmpA. We observed that, with increasing amounts of loaded protein, increased SipA was detected binding to the blot (Figure 3A). Importantly, the observed bands corresponded to the molecular weight of OmpA (35.5 kDa), which we could detect using an anti-FLAG antibody in the samples that were also subjected to far-western blotting (Supplementary Figure S2). We also observed faint bands corresponding to the size of OmpA in the wild type background. In addition, we detected bands of a lower molecular weight in both backgrounds. This could either represent a degradation product of OmpA that cannot be detected using the FLAG antibody or another potential interaction partner that remains to be identified.

To determine if OmpA interacts with SipA *in vivo*, we performed coimmunoprecipitation studies. His-tagged SipA was co-expressed in *V. cholerae* also containing a plasmid expressing FLAG-tagged OmpA. As a control, we also performed the coimmunoprecipitation in a *sipA* deletion strain. Cellular proteins were crosslinked using DSP, and the cells were lysed and applied to a nickel-NTA column. Prior to loading onto the column, both strains produced detectable OmpA and only the SipA-expressing strain produced SipA (input; Figure 3B). In the eluate from the column (output), OmpA was only detected in the strain expressing SipA, suggesting that the two proteins interact in the bacterial cell. OmpA was not detected in the eluate from the control, indicating that OmpA was binding to SipA and was not binding non-specifically to the Ni-NTA resin (Figure 3B). These data suggest that OmpA is an interaction partner of SipA and provides additional evidence that there may be a functional connection between this subfamily of proteins and OMPs.

### SipA and OmpA Likely Function in the Same Biological Pathway

**FIGURE 4:**
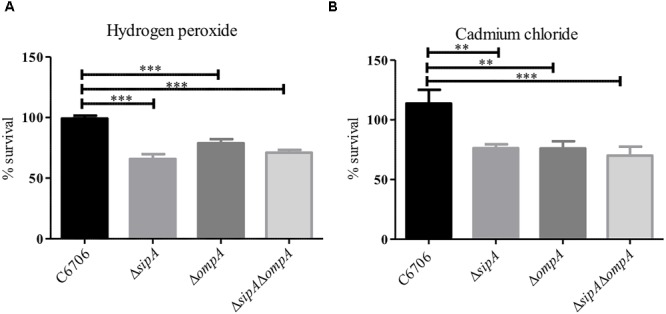
SipA and OmpA likely function in the same pathway. C6706, *sipA* mutant, *ompA* mutant, and a *sipA ompA* double mutant were grown to mid-logarithmic phase and treated with either **(A)** 2 μM hydrogen peroxide for 15 min or **(B)** 150 μM cadmium chloride for 45 min. After treatment, serial dilutions of each culture were plated for enumeration. Data represents the means and standard deviations of at least three samples. ^∗∗^*p* = < 0.01, ^∗∗∗^*p* < 0.001.

Based on our observation that SipA and OmpA interact in *V. cholerae*, we wanted to determine if OmpA contributes to the stress resistance phenotypes we had observed for SipA. We have already shown that a strain lacking *sipA* is sensitive to hydrogen peroxide and cadmium chloride (Figure 1). We predicted that, if OmpA contributes to hydrogen peroxide and cadmium chloride stress relief in conjunction with SipA as part of a shared mechanism, an *ompA* mutant would also be similarly sensitive to both compounds. To that end, we generated an *ompA* deletion strain and assayed its survival in the presence of hydrogen peroxide and cadmium chloride (Figure 4). We found that the *ompA* deletion showed similar sensitivity as a *sipA* deletion. Additionally, one would expect that, if the two genes function in the same pathway, a double deletion would neither show an additive phenotype, nor significantly reduced survival. When we performed survival assays in a *V. cholerae* strain lacking both *sipA* and *ompA* we did not observe any further sensitivity to either compound (Figure 4). These results suggest that SipA and OmpA likely function in the same stress resistance pathway.

### SipA Interacts With Antimicrobial Peptides

**FIGURE 5:**
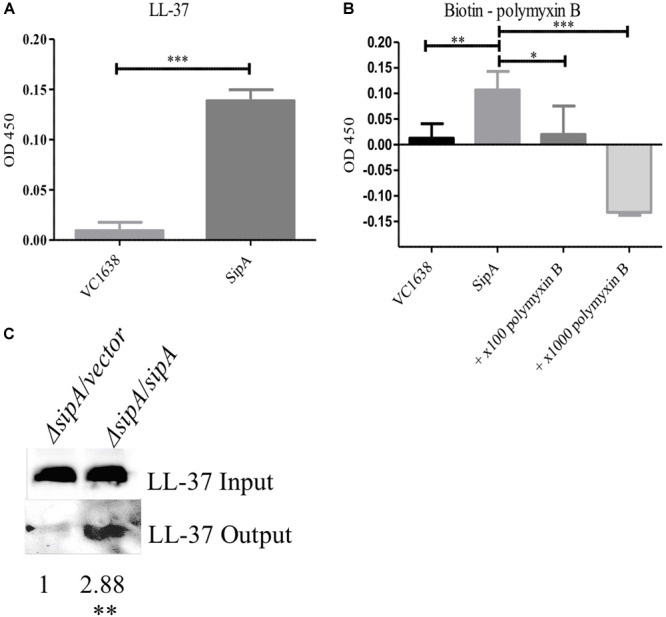
SipA binds polymyxin B and LL-37 *in vitro* and *in vivo*. Wells from a 96-well plate were coated with either purified SipA or VC1638 **(A,B)**. LL-37 **(A)** or biotin-labeled polymyxin B **(B)** were added to plates and incubated for 1 h. Levels of binding were detected via **(A)** anti-LL-37 antibody followed by IgG-HRP or **(B)** Streptavidin-HRP. VC1638 served as a control protein. **(C)** Coimmunoprecipitation of LL-37 in the presence and absence of 6×His-*sipA*. Each culture was crosslinked and lysed before loading onto a Ni-NTA column (input), followed by elution with imidazole (output). Blots were probed with anti-LL-37 antibody. Densitometry of the LL-37 band was calculated based on three separate experiments and normalized to the strain containing empty vector. ^∗^*p* = < 0.05, ^∗∗^*p* = < 0.01, ^∗∗∗^*p* = < 0.001.

The function of the SipA homologs YdeI and YgiW is not fully understood, however, it has been hypothesized that YdeI could act as a chaperone that shuttles AMPs to the OMP OmpD for removal from the cell in *S.* Typhimurium (Pilonieta et al., 2009). However, interaction between AMPs and the SipA homologs has not been demonstrated previously. To determine if SipA could perform such a function, we tested whether or not SipA directly interacts with AMPs. We first tested this possibility *in vitro* using an enzyme-linked immunosorbent assay (ELISA) (Lin et al., 2015). To that end, His-tagged SipA was purified and used to coat wells of a 96-well plate. Then, either LL-37 or biotinylated polymyxin B was added. Antibodies were used to detect LL-37 (HRP conjugated) or biotin-labeled polymyxin B (Streptavidin-HRP). As a negative control, we also assessed AMP binding to VC1638, a protein that is not predicted to interact with AMPs. Significantly higher binding of both LL-37 and polymyxin B to SipA was observed compared to the control protein (Figure 5A,B). In addition, due to the fact that the polymyxin B was biotin-labeled for detection purposes, we were also able to test whether the addition of excess unlabeled polymyxin specifically competed with the labeled polymyxin B for SipA binding (Figure 5B). The addition of unlabeled polymyxin B eliminated the binding of the biotin-labeled polymyxin B, indicating that the interaction observed between polymyxin B and SipA was direct and not due to the biotin label.

To further examine the interaction between SipA and AMPs we performed coimmunoprecipitation studies in bacterial cells. *sipA* mutant *V. cholerae* either containing an empty vector or a *sipA* overexpression plasmid was grown in the presence of LL-37. Cellular proteins were crosslinked, and the amount of LL-37 associated with the bacteria was assessed by immunoblotting. We observed an equivalent amount of LL-37 in the whole cell lysates prior to loading onto the nickel-NTA resin (input, Figure 5C). After bound proteins were eluted from the nickel column, we detected a much larger amount of LL-37 in the eluate from the SipA-expressing sample compared to *ΔsipA* strain (Figure 5C). This suggests that LL-37 can also interact with SipA *in vivo*. This is the first evidence that members of this family of proteins bind to AMPs.

**FIGURE 6:**
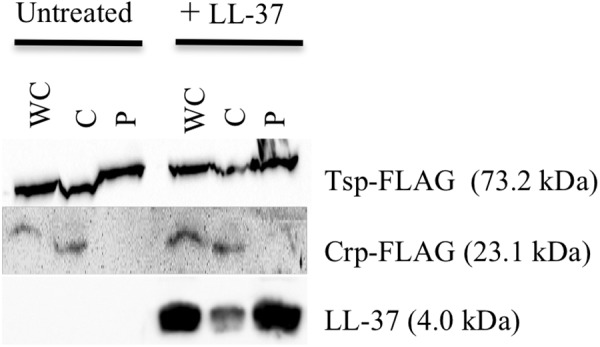
LL-37 is found in the periplasm of *V. cholerae*. Immunoblotting of whole-cell lysates (WC) and soluble fractions from C6707 grown in the presence and absence of LL-37. The membranes were probed with anti-FLAG or anti-LL-37 antibodies. Tsp-FLAG and Crp-FLAG were used as markers for the periplasmic (P) and cytoplasmic (C) fractions, respectively.

For the observed interaction between LL-37 and SipA to be biologically relevant, it would require that AMPs gain access to the periplasmic space of *V. cholerae*, where SipA is likely to perform its cellular function. Previous studies showed that LL-37 can enter the periplasm of *E. coli* (Sochacki et al., 2011), but this had not been tested in *V. cholerae*. After treatment of bacteria with a sublethal concentration of LL-37 and fractionating into soluble compartments, LL-37 was detected in both the cytoplasmic and the periplasmic space (Figure 6). This suggests that LL-37 is able to enter the periplasm, the likely location in which the interaction between AMPs and SipA occurs.

### SipA Is Required for AMP Resistance in Classical *V. cholerae*

**FIGURE 7:**
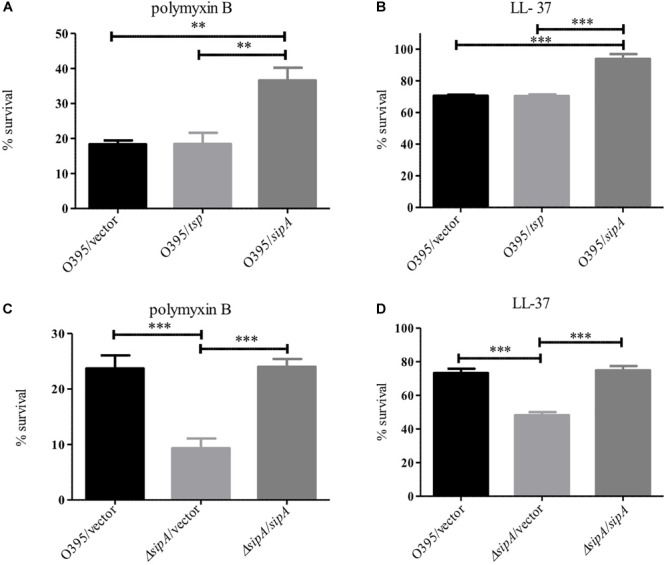
SipA is required for AMP resistance in the Classical biotype of *V. cholerae*. O395 (Classical biotype) overexpressing Tsp or SipA **(A,B)**, and an isogenic *sipA* mutant **(C,D)** were treated with 10 μg/mL polymyxin B **(A,C)** or 5 μg/mL LL-37 **(B,D)**. After treatment, serial dilutions of each culture were plated for enumeration. Data represents the means and standard deviations of at least three samples. ^∗∗^*p* = < 0.01, ^∗∗∗^*p* = < 0.001.

One of the more confounding observations from our previous studies on SipA was that a deletion strain did not show increased sensitivity to polymyxin B, in spite of its expression being highly induced by this stress (Matson et al., 2017). However, all of our studies to date have investigated the role of SipA in the El Tor biotype, which is highly resistant to polymyxin B (Roy et al., 1965; Henderson et al., 2017). Classical strains of *V. cholerae* are more sensitive to AMPs due to a frameshift mutation in *almF*, a gene in a lipid A modification pathway that is functional in strains of the El Tor biotype (Matson et al., 2010; Samanta et al., 2015). Therefore, we determined if the role of SipA in AMP resistance was more important in this less-resistant background. We first assessed survival of Classical (O395) *V. cholerae* in the presence of polymyxin B and LL-37 when SipA was overexpressed from a plasmid (Figure 7A,B). The wild type strain (containing empty vector) showed approximately 20% survival in the presence of 10 μg/mL polymyxin B and 70% survival in 5 μg/mL LL-37 after 1 h. Comparatively, a strain overexpressing SipA showed significantly increased survival, approximately twofold in polymyxin B and 1.4-fold in LL-37 (Figure 7A,B). As a control, the periplasmic protein Tsp was also overexpressed and did not increase survival. This suggests that the presence of increased levels of SipA protects *V. cholerae* against AMP killing. To further test the role of SipA in AMP resistance, we generated a *sipA* mutant in the Classical background of *V. cholerae* (strain O395). When the mutant was exposed to polymyxin B or LL-37 it showed a defect in survival that could be restored by complementing with *sipA* expressed from a plasmid (Figure 7C,D). In order to determine if OmpA also plays a role in AMP resistance in this background, we generated an *ompA* mutant and tested its ability to survive in the presence of polymyxin B. Similar to a *sipA* mutant, the strain lacking *ompA* shows a survival defect in the presence of AMPs (Supplementary Figure S3). These results demonstrate that SipA plays a role in *V. cholerae* surviving AMP stress, and that this role is masked in the highly resistant El Tor background.

## Discussion

Many mechanisms of resistance to AMP stress have been previously described in Gram-negative bacteria, including *V. cholerae*. Despite years of study on this type of stress response, we are still identifying new ways that bacteria respond to insults to their cell envelope. In this study, we describe a new function for a member of the OB-fold family of proteins. While SipA homologs have been shown previously to contribute to AMP, cadmium chloride, and hydrogen peroxide stress (Pilonieta et al., 2009; Lee et al., 2010), the mechanism of stress relief was unclear. The homologs were shown to interact with various porins, and we similarly found that SipA interacts with OmpA in *V. cholerae* (Figure 3). However, one of the most novel findings of this study was the discovery that SipA interacts with AMPs (Figure 5). This is the first time that a member of the OB-fold family of proteins has been shown to bind AMPs directly, shedding more light on possible functional roles for these proteins.

**FIGURE 8:**
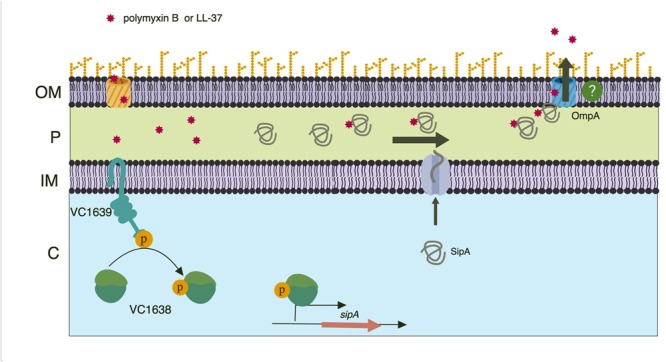
Model of SipA-mediated relief from AMP stress. A two-component system, VC1638/39, activates expression of *sipA* in response AMP exposure. Once made, the SipA protein localizes to the periplasm where it binds AMPs that have entered the bacterial cell. SipA then transports the AMPs to the outer membrane porin OmpA, with which it also interacts. Through an unclear mechanism, the AMPs then exit the cell. While this mechanism is present in both the Classical and El Tor strains of *V. cholerae*, we speculate that AMPs are less likely to enter the bacterial cell in El Tor strains due to the presence of an intact lipid A modification system. Model created using BioRender.

From our data we predict that SipA/OmpA has a role similar to that of YgiW/OmpF and YdeI/OmpD (Pilonieta et al., 2009); namely that they function together to protect bacteria against cationic AMPs. In addition, we predict that this subfamily of proteins interacts with AMPs in the bacterial periplasmic space. We therefore propose a model where, upon AMP exposure, the VC1638/39 TCS activates expression of *sipA*. Once made, the SipA protein localizes to the periplasm where it interacts with AMPs that have gained access to the cellular compartment. We also suggest that SipA chaperones the AMPs to OmpA, positioning them for efflux out of the cell (Figure 8). How SipA binds and possibly then releases AMPs is not clear. One could speculate that SipA remains bound to the AMP and exits the cell through OmpA. OmpA is predicted to have a pore size of 1 to 2.4 nm in *E. coli* (Sugawara and Nikaido, 1992; Sun et al., 2013). If *V. cholerae* OmpA has a larger pore size, it is possible that SipA could leave the periplasm in complex with the AMPs. Alternatively, there may be a mechanism by which SipA disassociates with its cargo and remains in the periplasm. This seems more efficient, as unbound SipA would then be able to continue to perform its function if AMPs continue to accumulate in the periplasm. Another possible way that SipA and OmpA could act together to contribute to AMP stress relief is by SipA blocking influx of AMPs into the cell through the OmpA pore. It is possible that SipA interacts with OmpA near the pore, preventing entry of AMPs into the periplasm through the pore by acting as a molecular plug (van der Heijden et al., 2016). Additional studies are needed to determine in more detail how these two proteins act to relieve AMP stress in *V. cholerae* and if these functions are conserved in its homologs.

How SipA and its homologs relieve cadmium chloride and hydrogen peroxide stress is less clear. It is likely that relief from these two stressors uses SipA in a different way than AMPs, as these small molecules seem less likely to directly bind the protein. OmpA appears to play a role in this stress relief pathway as well, as both a single and double mutant (with *sipA*) show similarly decreased survival in the presence of these stressors. How OmpA works in conjunction with SipA to either rid the periplasm of these compounds, or act to prevent their entry, is unclear.

While cadmium chloride and hydrogen peroxide induce expression of *sipA* (Supplementary Figure S1), we do not know if that induction requires the VC1638/39 TCS. It is possible that another regulatory network stimulates *sipA* expression in the presence of other stressors. Of course, we still may identify other stress-inducing agents that require SipA for relief, which may shed more light onto a common mechanistic theme for this pathway.

While we have gained more insight into the function of the SipA protein, there are still additional questions on the upstream regulation of *sipA* expression. We have demonstrated that the VC1638/39 TCS is required for *sipA* expression (Matson et al., 2017), but it is not known if VC1638 directly binds to the *sipA* promoter, activating its expression, or if there are additional or intermediary transcriptional activators involved. Additionally, we do not know if expression of *vc1638/39* is conditionally regulated or if it is constitutively expressed. Unlike *sipA*, our transcriptomic studies showed that polymyxin B exposure did not significantly change expression of either gene of the TCS (Matson et al., 2017). However, it does appear that *sipA* may be a unique regulatory target of the TCS, as there were no other genes whose expression was induced by polymyxin B and dependent on the presence of the TCS.

One of the most confounding observations going into this study was that a *sipA* mutant strain did not have a survival defect when exposed to polymyxin B, in spite of its expression being highly induced by AMPs. However, prior to the present study we had only investigated the role of SipA in the highly AMP resistant El Tor biotype of *V. cholerae* (Henderson et al., 2014; Matson et al., 2017). When we tested survival in the Classical biotype, which cannot modify its lipid A, we found that SipA contributes to bacterial survival in the presence of both polymyxin B and LL-37 (Figure 7). This suggests that lipid A modification acts as a successful first line of defense against AMPs, and likely prevents a large amount of AMP entry into the cell. In the absence of such modification, it is likely that more AMPs enter the cell. In this event, SipA appears to act as an important second line of defense, aiding in the removal of AMPs from the periplasm.

In the Classical biotype survival assays, a *sipA* mutant shows decreased survival, but a fraction of the population still survives. We therefore hypothesize that in the absence of lipid A modification and *sipA*, there are yet other mechanisms that protect the bacteria from AMP killing. Gram-negative bacteria have numerous mechanisms to counteract the negative effects of AMPs on cellular functions, and some of these may contribute to survival in the absence of these primary strategies. One mechanism could be through the production of OMVs. OMVs are produced constitutively by Gram-negative bacteria and aid in the elimination of undesired compounds from the cell (McBroom and Kuehn, 2007; Manning and Kuehn, 2011; Schwechheimer and Kuehn, 2013, 2015; Jan, 2017). Additionally, OMVs have a defined role in protection of Gram-negative bacteria against antibiotics and antimicrobials (Kulkarni et al., 2015). Another method of AMP resistance may be their removal through resistance-nodulation-division (RND) efflux systems. These systems work by decreasing the permeability of the outer membrane and regulating efflux of antimicrobials out of the cell. In the absence of RND efflux systems, El Tor strains of *V. cholerae* become 5-fold more sensitive to polymyxin B (Bina et al., 2008) indicating its importance to antimicrobial resistance and suggesting that this may be another layer of protection against AMPs.

Collectively, these observations illustrate the multiple layers of resistance against AMPs in Gram-negative bacteria, and suggest a new resistance mechanism in *V. cholerae* that involves interactions between an OB-fold protein, AMPs, and porins. Inhibition of these systems may have therapeutic benefit, by sensitizing bacteria to host innate immune defenses.

## Author Contributions

JS-M and JM conceived and designed the study, wrote the manuscript, and analyzed the data. JS-M performed all the experiments.

## Conflict of Interest Statement

The authors declare that the research was conducted in the absence of any commercial or financial relationships that could be construed as a potential conflict of interest.

## References

[B1] Baba T. Ara T. Hasegawa M. Takai Y. Okumura Y. Baba M. (2006). Construction of *Escherichia coli* K-12 in-frame, single-gene knockout mutants: the Keio collection. *Mol. Syst. Biol.* 2:0008. 10.1038/msb4100050 16738554 PMC1681482

[B2] Bahar A. A. Ren D. (2013). Antimicrobial peptides. *Pharmaceuticals* 6 1543–1575. 10.3390/ph6121543 24287494 PMC3873676

[B3] Band V. I. Weiss D. S. (2015). Mechanisms of antimicrobial peptide resistance in gram-negative bacteria. *Antibiotics* 4 18–41. 10.3390/antibiotics4010018 25927010 PMC4410734

[B4] Beck N. A. Krukonis E. S. DiRita V. J. (2004). TcpH influences virulence gene expression in *Vibrio cholerae* by inhibiting degradation of the transcription activator TcpP. *J. Bacteriol.* 186 8309–8316. 10.1128/JB.186.24.8309-8316.2004 15576780 PMC532408

[B5] Bilecen K. Fong J. C. Cheng A. Jones C. J. Zamorano-Sanchez D. Yildiz F. H. (2015). Polymyxin B resistance and biofilm formation in *Vibrio cholerae* are controlled by the response regulator CarR. *Infect. Immun.* 83 1199–1209. 10.1128/IAI.02700-14 25583523 PMC4333464

[B6] Bina X. R. Provenzano D. Nguyen N. Bina J. E. (2008). *Vibrio cholerae* RND family efflux systems are required for antimicrobial resistance, optimal virulence factor production, and colonization of the infant mouse small intestine. *Infect. Immun.* 76 3595–3605. 10.1128/IAI.01620-07 18490456 PMC2493215

[B7] Brownlee G. Jones T. S. (1948). The polymyxins; a related series of antibiotics derived from B. *polymyxa*. *Biochem. J.* 43:25. 18208231

[B8] Carlone G. M. Thomas M. L. Rumschlag H. S. Sottnek F. O. (1986). Rapid microprocedure for isolating detergent-insoluble outer membrane proteins from Haemophilus species. *J. Clin. Microbiol.* 24 330–332. 3489731 10.1128/jcm.24.3.330-332.1986 PMC268907

[B9] Choi H. Yang Z. Weisshaar J. C. (2017). Oxidative stress induced in *E. coli* by the human antimicrobial peptide LL-37. *PLoS Pathog.* 13:e1006481. 10.1371/journal.ppat.1006481 28665988 PMC5509375

[B10] Durr U. H. Sudheendra U. S. Ramamoorthy A. (2006). LL-37, the only human member of the cathelicidin family of antimicrobial peptides. *Biochim. Biophys. Acta* 1758 1408–1425. 10.1016/j.bbamem.2006.03.030 16716248

[B11] Ginalski K. Kinch L. Rychlewski L. Grishin N. V. (2004). BOF: a novel family of bacterial OB-fold proteins. *FEBS Lett.* 567 297–301. 10.1016/j.febslet.2004.04.086 15178340

[B12] Guzman L. M. Belin D. Carson M. J. Beckwith J. (1995). Tight regulation, modulation, and high-level expression by vectors containing the arabinose PBAD promoter. *J. Bacteriol.* 177 4121–4130. 10.1128/jb.177.14.4121-4130.1995 7608087 PMC177145

[B13] Henderson J. C. Fage C. D. Cannon J. R. Brodbelt J. S. Keatinge-Clay A. T. Trent M. S. (2014). Antimicrobial peptide resistance of *Vibrio cholerae* results from an LPS modification pathway related to nonribosomal peptide synthetases. *ACS Chem. Biol.* 9 2382–2392. 10.1021/cb500438x 25068415 PMC4520716

[B14] Henderson J. C. Herrera C. M. Trent M. S. (2017). AlmG, responsible for polymyxin resistance in pandemic *Vibrio cholerae*, is a glycyltransferase distantly related to lipid a late acyltransferases. *J. Biol. Chem.* 292 21205–21215. 10.1074/jbc.RA117.000131 29101229 PMC5743092

[B15] Herrera C. M. Crofts A. A. Henderson J. C. Pingali S. C. Davies B. W. Trent M. S. (2014). The *Vibrio cholerae* VprA-VprB two-component system controls virulence through endotoxin modification. *mBio* 5:e2283-14. 10.1128/mBio.02283-14 25538196 PMC4278540

[B16] Herrera C. M. Hankins J. V. Trent M. S. (2010). Activation of PmrA inhibits LpxT-dependent phosphorylation of lipid a promoting resistance to antimicrobial peptides. *Mol. Microbiol.* 76 1444–1460. 10.1111/j.1365-2958.2010.07150.x 20384697 PMC2904496

[B17] Huntley J. F. Conley P. G. Hagman K. E. Norgard M. V. (2007). Characterization of francisella tularensis outer membrane proteins. *J. Bacteriol.* 189 561–574. 10.1128/JB.01505-06 17114266 PMC1797401

[B18] Innis M. A. (1990). *PCR protocols : a guide to methods and applications.* San Diego, CA: Academic Press.

[B19] Izadpanah A. Gallo R. L. (2005). Antimicrobial peptides. *J. Am. Acad. Dermatol.* 52(3 Pt 1) 381–390; quiz 391–382. 10.1016/j.jaad.2004.08.026 15761415

[B20] Jan A. T. (2017). Outer Membrane Vesicles (OMVs) of gram-negative bacteria: a perspective update. *Front. Microbiol.* 8:1053. 10.3389/fmicb.2017.01053 28649237 PMC5465292

[B21] Joelsson A. Kan B. Zhu J. (2007). Quorum sensing enhances the stress response in *Vibrio cholerae*. *Appl. Environ. Microbiol.* 73 3742–3746. 10.1128/AEM.02804-06 17434996 PMC1932696

[B22] Kulkarni H. M. Nagaraj R. Jagannadham M. V. (2015). Protective role of *E. coli* outer membrane vesicles against antibiotics. *Microbiol. Res.* 181 1–7. 10.1016/j.micres.2015.07.008 26640046

[B23] Lee J. Hiibel S. R. Reardon K. F. Wood T. K. (2010). Identification of stress-related proteins in *Escherichia coli* using the pollutant cis-dichloroethylene. *J. Appl. Microbiol.* 108 2088–2102. 10.1111/j.1365-2672.2009.04611.x 19919618

[B24] Lin M. F. Tsai P. W. Chen J. Y. Lin Y. Y. Lan C. Y. (2015). OmpA binding mediates the effect of antimicrobial peptide LL-37 on *Acinetobacter baumannii*. *PLoS One* 10:e0141107. 10.1371/journal.pone.0141107 26484669 PMC4618850

[B25] Linn T. St Pierre R. (1990). Improved vector system for constructing transcriptional fusions that ensures independent translation of lacZ. *J. Bacteriol.* 172 1077–1084. 10.1128/jb.172.2.1077-1084.1990 2137119 PMC208539

[B26] Manning A. J. Kuehn M. J. (2011). Contribution of bacterial outer membrane vesicles to innate bacterial defense. *BMC Microbiol.* 11:258. 10.1186/1471-2180-11-258 22133164 PMC3248377

[B27] Matson J. S. DiRita V. J. (2005). Degradation of the membrane-localized virulence activator TcpP by the YaeL protease in *Vibrio cholerae*. *Proc. Natl. Acad. Sci. U.S.A.* 102 16403–16408. 10.1073/pnas.0505818102 16254052 PMC1283431

[B28] Matson J. S. Livny J. DiRita V. J. (2017). A putative *Vibrio cholerae* two-component system controls a conserved periplasmic protein in response to the antimicrobial peptide polymyxin B. *PLoS One* 12:e0186199. 10.1371/journal.pone.0186199 29020117 PMC5636140

[B29] Matson J. S. Yoo H. J. Hakansson K. Dirita V. J. (2010). Polymyxin B resistance in El Tor *Vibrio cholerae* requires lipid acylation catalyzed by MsbB. *J. Bacteriol.* 192 2044–2052. 10.1128/JB.00023-10 20154134 PMC2849451

[B30] McBroom A. J. Kuehn M. J. (2007). Release of outer membrane vesicles by gram-negative bacteria is a novel envelope stress response. *Mol. Microbiol.* 63 545–558. 10.1111/j.1365-2958.2006.05522.x 17163978 PMC1868505

[B31] Miller J. H. (1972). *Experiments in molecular genetics.* Cold Spring Harbor, NY: Cold Spring Harbor Laboratory Press.

[B32] Nizet V. (2006). Antimicrobial peptide resistance mechanisms of human bacterial pathogens. *Curr. Issues Mol. Biol.* 8 11–26. 16450883

[B33] Petiti M. Houot L. Duche D. (2017). Cell fractionation. *Methods Mol. Biol.* 1615 59–64. 10.1007/978-1-4939-7033-9_3 28667601

[B34] Pilonieta M. C. Erickson K. D. Ernst R. K. Detweiler C. S. (2009). A protein important for antimicrobial peptide resistance, YdeI/OmdA, is in the periplasm and interacts with OmpD/NmpC. *J. Bacteriol.* 191 7243–7252. 10.1128/JB.00688-09 19767429 PMC2786563

[B35] Roy C. Mridha K. Mukerjee S. (1965). Action of Polymyxin of cholera vibrios. techniques of determination of polymyxin-sensitivity. *Proc. Soc. Exp. Biol. Med.* 119 893–896. 10.3181/00379727-119-30329 14329028

[B36] Samanta P. Ghosh P. Chowdhury G. Ramamurthy T. Mukhopadhyay A. K. (2015). Sensitivity to polymyxin B in El Tor *Vibrio cholerae* O1 strain, Kolkata, India. *Emerg. Infect. Dis.* 21 2100–2102. 10.3201/eid2111.150762 26488385 PMC4622255

[B37] Schwechheimer C. Kuehn M. J. (2013). Synthetic effect between envelope stress and lack of outer membrane vesicle production in *Escherichia coli*. *J. Bacteriol.* 195 4161–4173. 10.1128/JB.02192-12 23852867 PMC3754735

[B38] Schwechheimer C. Kuehn M. J. (2015). Outer-membrane vesicles from gram-negative bacteria: biogenesis and functions. *Nat. Rev. Microbiol.* 13 605–619. 10.1038/nrmicro3525 26373371 PMC5308417

[B39] Skorupski K. Taylor R. K. (1996). Positive selection vectors for allelic exchange. *Gene* 169 47–52. 10.1016/0378-1119(95)00793-8 8635748

[B40] Sochacki K. A. Barns K. J. Bucki R. Weisshaar J. C. (2011). Real-time attack on single *Escherichia coli* cells by the human antimicrobial peptide LL-37. *Proc. Natl. Acad. Sci. U.S.A.* 108 E77–E81. 10.1073/pnas.1101130108 21464330 PMC3080975

[B41] Sorensen O. E. Borregaard N. Cole A. M. (2008). Antimicrobial peptides in innate immune responses. *Contrib. Microbiol.* 15 61–77. 10.1159/000136315 18511856

[B42] Sugawara E. Nikaido H. (1992). Pore-forming activity of OmpA protein of *Escherichia coli*. *J. Biol. Chem.* 267 2507–2511. 1370823

[B43] Sun D. Wang B. Zhu L. Chen M. Zhan L. (2013). Block and boost DNA transfer: opposite roles of OmpA in natural and artificial transformation of *Escherichia coli*. *PLoS One* 8:e59019. 10.1371/journal.pone.0059019 23533598 PMC3606455

[B44] Teoh W. P. Matson J. S. DiRita V. J. (2015). Regulated intramembrane proteolysis of the virulence activator TcpP in Vibrio cholerae is initiated by the tail-specific protease (Tsp). *Mol. Microbiol.* 97 822–831. 10.1111/mmi.13069 25999037

[B45] van der Heijden J. Reynolds L. A. Deng W. Mills A. Scholz R. Imami K. (2016). *Salmonella* rapidly regulates membrane permeability to survive oxidative stress. *mBio* 7:e1238-16. 10.1128/mBio.01238-16 27507830 PMC4992977

[B46] Wu Y. Li Q. Chen X. Z. (2007). Detecting protein-protein interactions by far western blotting. *Nat. Protoc.* 2 3278–3284. 10.1038/nprot.2007.459 18079728

