# Supplementary material for: A Periplasmic Antimicrobial Peptide-Binding Protein Is Required for Stress Survival in Vibrio cholerae
Source: Front Microbiol. 2019 Feb 5;10:161. doi: 10.3389/fmicb.2019.00161 (PMC6370654; doi:10.3389/fmicb.2019.00161)
Supplement: Supplementary file 1 [file Table_1.docx]

**Table S1.** Strains and plasmids used in this study.

| Strains | Source or Reference |
| --- | --- |
| *Vibrio cholerae* |  |
| O395 (Classical *V. cholerae*) | Laboratory collection |
| C6706 (El Tor *V. cholerae*) | Laboratory collection |
| C6706 Δ*sipA* | 18 |
| C6706 *sipA*-6xHis | This study |
| C6706 Δ*ompA* | This study |
| C6706 Δ*ompA* Δ*sipA* | This study |
| O395 Δ*sipA* | This study |
| O395 Δ*ompA* | This study |
| *Escherichia coli* |  |
| DH5α | Laboratory collection |
| JM101 | Laboratory collection |
| DH5α λpir | Laboratory collection |
| SM10 λpir | Laboratory collection |
| K-12 BW25113 | 34 |
| K-12 BW25113 Δ*ygiW* | 34 |
| Plasmids |  |
| pBAD18-Kan | 20 |
| pBAD33 | 20 |
| pKAS32 | 21 |
| pTL61T | 22 |
| pTL61T + *sipA*pro (0732pro) | 18 |
| pKAS32 + Δ*sipA* | 18 |
| pKAS32 + Δ*ompA* | This study |
| pBAD18-Kan + *tsp*-FLAG | This study |
| pBAD18-Kan + *sipA*-6xHis | This study |
| pBAD18-Kan + *ygiW* | This study |
| pBAD33 + *crp*-FLAG | This study |
| pBAD18-Kan + *ompA*-FLAG | This study |
