# Supplementary material for: A Periplasmic Antimicrobial Peptide-Binding Protein Is Required for Stress Survival in Vibrio cholerae
Source: Front Microbiol. 2019 Feb 5;10:161. doi: 10.3389/fmicb.2019.00161 (PMC6370654; doi:10.3389/fmicb.2019.00161)
Supplement: Supplementary file 2 [file Presentation_1.PPTX]

## Slide 1
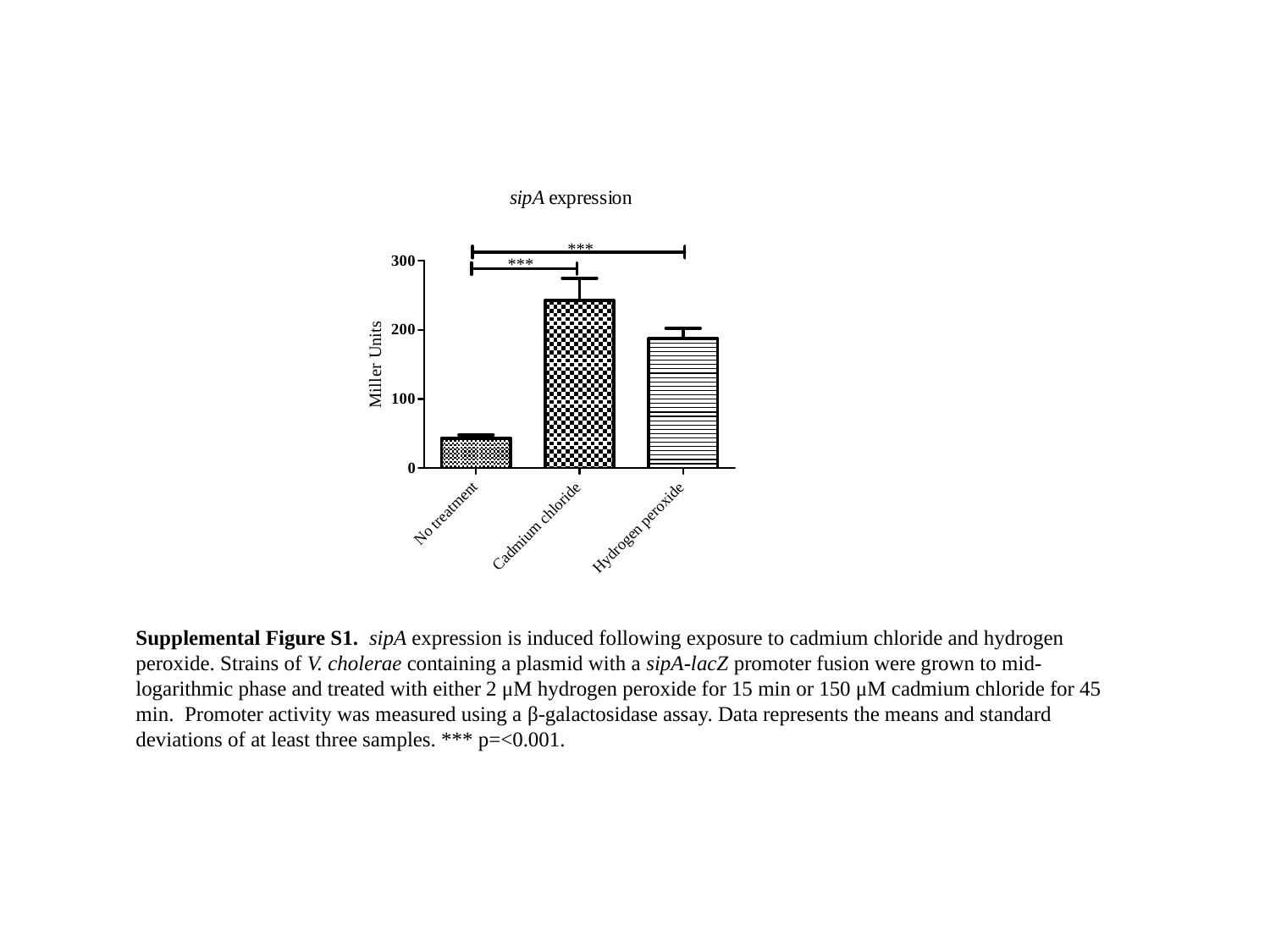

Supplemental Figure S1. sipA expression is induced following exposure to cadmium chloride and hydrogen peroxide. Strains of V. cholerae containing a plasmid with a sipA-lacZ promoter fusion were grown to mid-logarithmic phase and treated with either 2 μM hydrogen peroxide for 15 min or 150 μM cadmium chloride for 45 min. Promoter activity was measured using a β-galactosidase assay. Data represents the means and standard deviations of at least three samples. *** p=<0.001.

## Slide 2
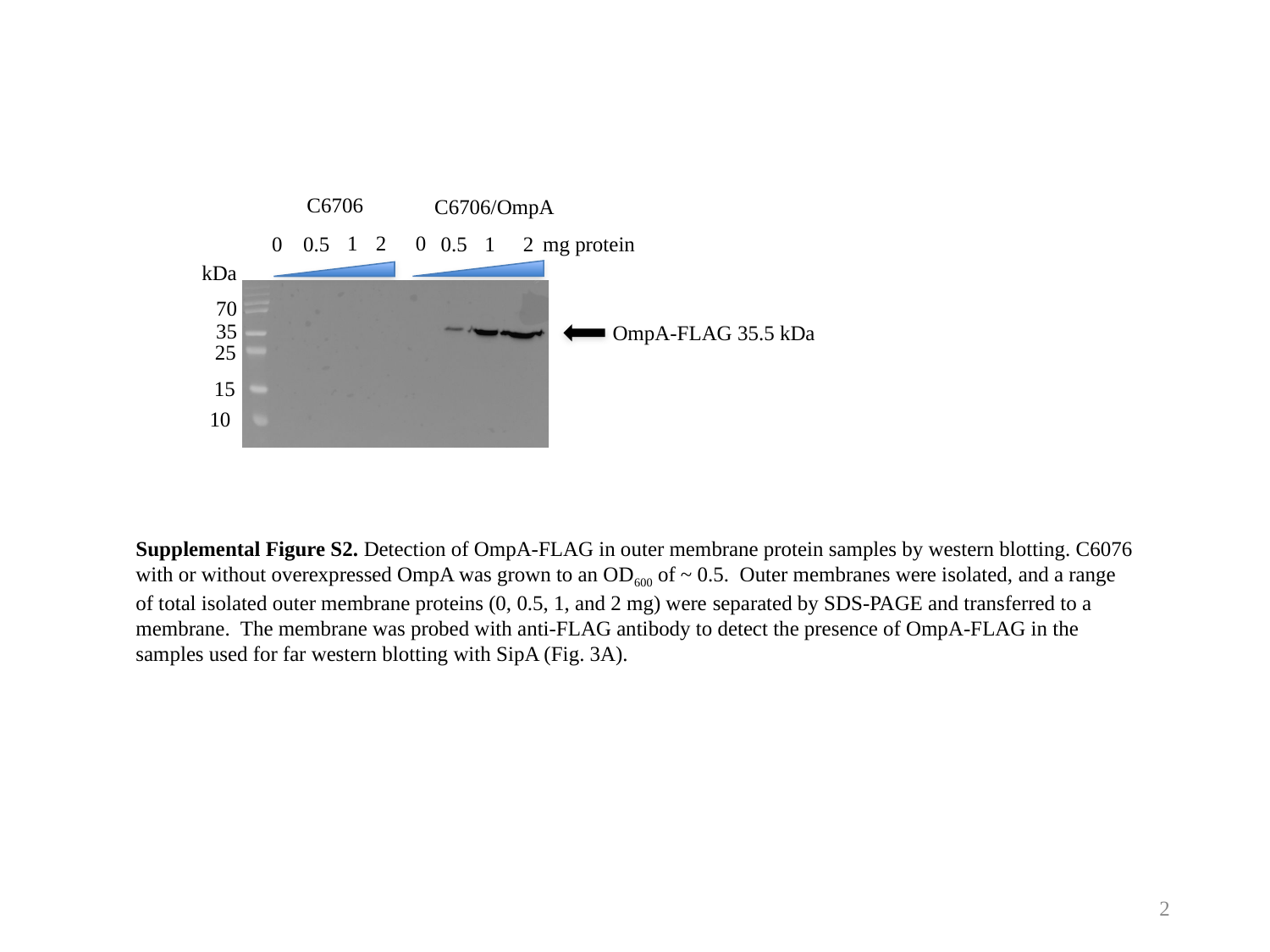

C6706
C6706/OmpA
1
2
0
0
0.5
2
mg protein
0.5
1
kDa
70
35
OmpA-FLAG 35.5 kDa
25
15
10
Supplemental Figure S2. Detection of OmpA-FLAG in outer membrane protein samples by western blotting. C6076 with or without overexpressed OmpA was grown to an OD600 of ~ 0.5. Outer membranes were isolated, and a range of total isolated outer membrane proteins (0, 0.5, 1, and 2 mg) were separated by SDS-PAGE and transferred to a membrane. The membrane was probed with anti-FLAG antibody to detect the presence of OmpA-FLAG in the samples used for far western blotting with SipA (Fig. 3A).
2

## Slide 3
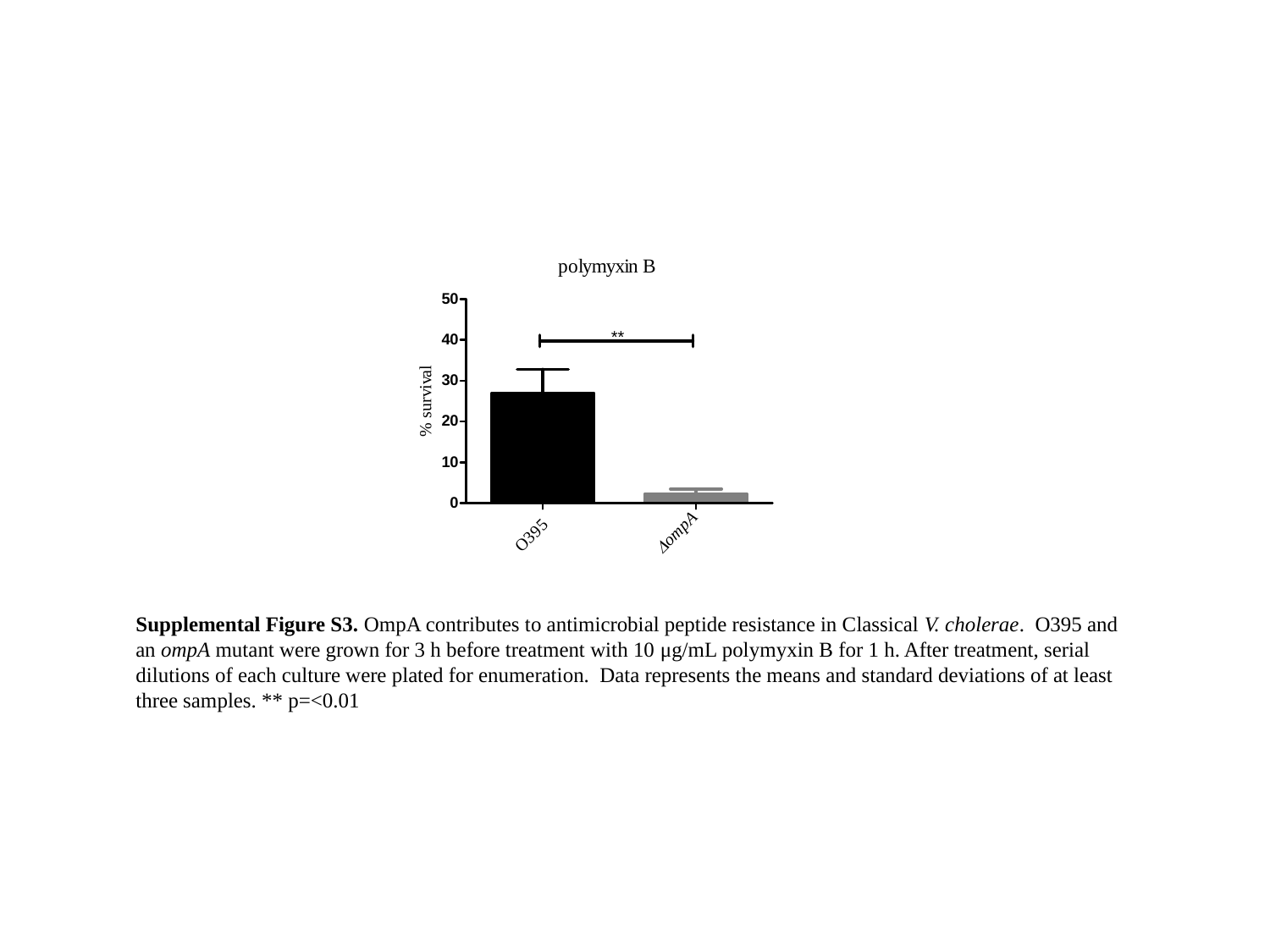

Supplemental Figure S3. OmpA contributes to antimicrobial peptide resistance in Classical V. cholerae. O395 and an ompA mutant were grown for 3 h before treatment with 10 μg/mL polymyxin B for 1 h. After treatment, serial dilutions of each culture were plated for enumeration. Data represents the means and standard deviations of at least three samples. ** p=<0.01
